# Kinetic Study on the Formation of Bimetallic Core-Shell Nanoparticles via Microemulsions

**DOI:** 10.3390/ma7117513

**Published:** 2014-11-21

**Authors:** Concha Tojo, Nuria Vila-Romeu

**Affiliations:** 1 Physical Chemistry Department, Faculty of Chemistry, University of Vigo, E-36310 Vigo, Spain; 2 Physical Chemistry Department, Faculty of Sciences, University of Vigo, E-32004 Ourense, Spain; E-Mail: nvromeu@uvigo.es

**Keywords:** bimetallic nanoparticles, microemulsion, intermicellar exchange rate

## Abstract

Computer calculations were carried out to determine the reaction rates and the mean structure of bimetallic nanoparticles prepared via a microemulsion route. The rates of reaction of each metal were calculated for a particular microemulsion composition (fixed intermicellar exchange rate) and varying reduction rate ratios between both metal and metal salt concentration inside the micelles. Model predictions show that, even in the case of a very small difference in reduction potential of both metals, the formation of an external shell in a bimetallic nanoparticle is possible if a large reactant concentration is used. The modification of metal arrangement with concentration was analyzed from a mechanistic point of view, and proved to be due to the different impact of confinement on each metal: the reaction rate of the faster metal is only controlled by the intermicellar exchange rate but the slower metal is also affected by a cage-like effect.

## 1. Introduction

Bimetallic nanoparticles, in which two kinds of metals are assembled, are particularly attractive because their properties often differ markedly from either of the constituent metals [1,2,3]. The presence of a second metal modifies the physical and chemical interactions, thus spatial distribution of atoms changes the chemical and physical properties of the nanoparticle [4,5]. Because the properties of bimetal nanoparticles strongly depend on their size, structure and morphology [6,7], the design and control of the spatial arrangement of both metals in bimetallic nanoparticles are critical for exploiting their potential applications [8]. Nowadays, much effort has been devoted to the preparation of bimetallic nanoparticles with controlled composition distribution [9,10,11,12,13].

Because the synthetic route seems to be crucial in determining the final metal distributions of bimetallic nanoparticles, our study was focused on a concrete method, the one-pot microemulsion technique. Microemulsions are thermodynamically stable dispersions in which two liquids initially immiscible (water and oil) coexist in one phase due to the presence of a monolayer of surfactant molecules. The main advantage of using microemulsions is the fact that the size of the resulting micelles can be controlled by varying the temperature and the composition of the microemulsion. The surfactant-stabilized droplets provide a microenvironment for the preparation of nanoparticles by exchanging their contents and preventing excess aggregation of particles. In addition, nanoparticles prepared in microemulsions often exhibit better surface properties, so nanocatalysts obtained from microemulsions show better activity and selectivity than those prepared by other methods [14]. For the synthesis of bimetallic nanoparticles each reactant (metal salts and the reducing agent) is solubilized in the aqueous phase of a microemulsion. Microemulsion droplets are subject to Brownian motion, and collisions between them are frequent, leading to the formation of transient dimers. The material intermicellar exchange during the dimer formation allows the reactants to be in contact. The reduction of metal ions and the subsequent nanoparticle formation result from mixing the microemulsions. The nanoparticles obtained in such a medium are very fine and monodisperse. However, controlling the bimetallic distribution is a very difficult task even when microemulsions are used.

In the synthesis of bimetallic nanoparticles, the nucleus develops in a particle by building up new layers, so that the order of deposition of the metals defines the resulting structure. Core-shell nanostructures consist of a shell of one type of atom surrounding a core of another, though there may be some mixing between the shells. This structure is the most common in a large variety of systems, such as Au-Ag nanoparticles. Mixed alloys may be either ordered or random, and the random mixing arrangement is common to many systems, for example Pt-Pd nanoparticles. At first, when the synthesis takes place in a homogeneous media, it is assumed that the ions with a higher reduction potential are reduced first [15,16,17,18,19,20,21], so the difference in the reduction potential of two metal ions is the main factor determining the final structure of the particles: when one of the reduction reactions is faster, the first nuclei are composed of the fastest reduction product. Because the slower reduction product appears later, the outer layers show a progressive enrichment in the slower one. This effect is more pronounced as the difference between both reduction rates increases. However, this is too simple a picture, because this explanation was extended to synthesis in microemulsions without taking into account the compartmentalization of the reaction media. In fact, a given bimetallic nanoparticle, such as Au-Ag [12,22] or Au-Pt [20,23], can be obtained in alloy form or in a core-shell structure depending on the microemulsion composition. This means that the rate of intermicellar exchange, which strongly depends on surfactant [24,25], plays an important role in the kinetics [26,27,28]. Previous simulation studies [29,30] concluded that the nanoparticle structure is defined by the difference in the reduction rates only if both reductions occur at the same rate (an alloy is obtained) or if both reductions have very different rates (a core-shell structure is obtained). These two extreme cases reproduce the behavior obtained in homogeneous media, that is, compartmentalization of the reaction media cannot modify the metal arrangement. However, a vast majority of bimetallic systems belong to the large space between both extremes (1 < v_A_/v_B_ < 100, where v_A_ and v_B_ are the reduction rates of fast and slow metal respectively), in which metal distribution depends on microemulsion composition. A more flexible surfactant allows a quicker exchange of reactants and an exchange of larger aggregates of products, favoring in this way the mixture of both metals at the atomic level [29].

However, the preparation of different nanostructures by changing the microemulsion composition (different surfactant, cosurfactant, *etc*.) gives rise to nanostructured particles with different cappings, which complicates the comparison of their properties. On the basis of the fact that a faster intermicellar exchange is able to diminish the natural metal nanosegregation when the difference in reduction rates is moderate [29], it was recently proved that metal arrangement can also be modified by changing only the reactant concentration [31]. Simulation results for a couple of metals characterized by a reduction rate ratio v_A_/v_B_ ≈ 10 and using a flexible surfactant film were successfully compared with Au/Pt nanoparticles synthesized using the same conditions of the simulation studies. A good agreement between theoretical and experimental STEM profiles confirmed the validity of the simulation model [31]. Based on this agreement, the model can be used as a tool to elucidate the complex interactions of the dynamics of the colloidal reaction medium and the precipitation reaction. We present a kinetic study by means of systematic Monte Carlo simulations showing that the reaction rate of the metals is deeply modified by the compartmentalization of the reaction media. Consequently, the interplay between compartmentalization and reactant concentration can induce changes in the sequence of metal deposition, even in the case of a very small difference in reduction rates, when a flexible film is used.

The ratio between the reduction rates of the fast (v_A_) and the slow (v_B_) metal is a parameter used in the simulation model to characterize the nature of the metals. The structure of the Au/Pt bimetallic system, whose difference in standard reduction potentials is Δε = 0.26 V, was proven to be satisfactorily reproduced by simulation by means of a reduction rates ratio v_Au_/v_Pt_≈ 10 [31]. The present study is focused on bimetallic pairs with a difference in standard reduction potentials smaller than Δε ≈ 0.15 V, such as Pt-Pd (Δε ≈ 0.11 V) or Ag-Pd (Δε ≈ 0.12 V), which can be simulated by a reduction rate ratio v_A_/v_B_ ≈ 5. Results can be generalized to other bimetallic couples whose Δε is in this range. The catalytic potential of Au-Pt and Pt-Pd bimetallic systems has been proved. Recently, Ag-Pd pairs were studied [32] because of their catalytic activity [33,34,35,36], as well as their high sensitivity as detectors of cysteine [37]. This study contributes to fundamental research concerning the understanding of microemulsion-based nanoparticle synthesis, and can open up a new way to synthesize bimetallic nanoparticles with *ad-hoc* controlled nanostructures.

## 2. Simulation Model

The main strategy of the one pot method for the synthesis of Au/Pt bimetallic nanoparticles via microemulsions consists of mixing three microemulsions, one containing each reactant (the two metal salts and reducing agent). After mixing, micelles move and collide, allowing reactants be in contact with each other due to material transfer between colliding droplets. It is assumed that this intermicellar exchange occurs when a collision between two micelles is able to merge the micelles and establish a water channel between them. When one of the two metal salts (PdCl_4_^−^ or PtCl_6_^−^ used to prepare Pd/Pt particles) and the reductor (e.g., hydrazine) are located in the same micelle, chemical reduction takes place inside the reverse micelle to obtain metal atoms (Pd or Pt). That is, the droplets of microemulsion are conceived as tiny compartments or nanoreactors. They are ideal templates for nanoparticle synthesis because they can isolate a particle obtained within a micelle from those in neighboring micelles, thus preventing particle aggregation. The model tries to simulate the kinetic course of the chemical reaction inside these nanoreactors. In order to study the metal distribution in the final nanoparticle, the order of metal reduction inside each micelle is stored, and analyzed at the end of the synthesis.

### 2.1. Reaction Media Description

The microemulsion structure is assumed to consist of spherical micelles in a continuous oil phase. To reproduce this heterogeneous media, the microemulsion is defined as a set of micelles randomly located on a three dimensional lattice. Each simulation run starts with three different sets of micelles randomly distributed: micelles carrying A salt (M-A), B salt (M-B) and reducing agent (M-R). The fraction of the volume occupied by micelles is a φ = 10%. Each micelle can act as a nanoreactor during nanoparticle synthesis, so although initially each microemulsion carries only one kind of reactant, as the synthesis takes place different species can coexist together inside a micelle: reactants (faster reduction metal salt A^+^, slower reduction metal salt B^+^ and reducing agent R), free metal products (A and B), and growing particles (aggregates composed of A and B atoms).

Micelles diffuse by performing random walks. In a previous algorithm used to simulate the preparation of simple nanoparticles (non bimetallic), micelles moved to the nearest neighbor sites by choosing at random the direction of the motion at each step. The length of each step was constant and equal to one length of lattice unit. This random walk was subject to the exclusion principle so that the trial movements resulting in micelles overlapping, were excluded. Cyclic boundary conditions were enforced at the ends of the lattice. Micelles collided when they occupied contiguous lattice sites and only binary collisions were considered. In order to save computation time, the model was improved by simulating the movement and collisions as follows: Two micelles chosen at random are allowed to collide (due to random motion). Because of collision, micelles fuse to form a short-lived dimer, so a water channel can be established between them [24], allowing the exchange of material contents. After collision, micelles redisperse. All collision are here assumed to be effective. Both ways of simulating the motion and collision lead to exactly the same results [38]. The second method was used to simulate bimetallic nanoparticle synthesis because it is less time consuming.

### 2.2. Microemulsion Composition

Microemulsions are colloidal dispersions in which two liquids, initially inmiscible (water and oil), coexist in one phase due to the presence of a monolayer of surfactant in the interphase. The flexibility of the surfactant film surrounding micelles is a parameter associated with the interfacial curvature, which depends on the interactions at both sides of the interface, and this is dictated by microemulsion composition, mainly by the surfactant. So the flexibility of the surfactant film surrounding micelles is directly related to the facility with which intermicellar channels can be established. The intermicellar exchange of material takes place through the intermicellar channel, so the kinetics of nanoparticle formation will strongly depend on the channel feature, which in turn depends on the microemulsion composition.

Two aspects must be taken into account in order to establish how surfactant film flexibility is included in the simulation model: First, the material exchange between micelles will only be possible if the dimer remains long enough. The longer two colliding micelles stay together, the greater the number of species can be exchanged during a collision. So dimer stability can be directly related to the intermicellar exchange rate. Second, the intermicellar channel diameter restricts the size of the particles capable of crossing the channel. It is also related to microemulsion composition, because the more flexible the film, the larger the channel size. That is, a flexible film allows the exchange of larger particles than that of a rigid film. The most important factor determining the intermicellar exchange of an isolated species, such as reactants and non-aggregated metallic atoms, is the dimer stability [39], because free species traverse the channel one by one. So in this case the channel size would not be important. On the contrary, the channel size is the most determining factor if the exchanged specie is a growing particle (an aggregate of metal atoms), which is exchanged as a whole. From this picture, the flexibility of the surfactant film is introduced in the model by means of two simulation parameters: the exchange parameter *k_ex_*, which dictates the exchange protocol of free species (see below), and the flexibility parameter *f*, which restricts the size of the exchanged particles. Both parameters must rise together, because a flexible film implies that a larger particle can be transferred (high *f*) and intermicellar exchange is faster (high *k_ex_*). Experimental results obtained in a rigid microemulsion, such as AOT/n-heptane/water, were successful compared to simulation data where flexibility is characterized by *f* = 5 and *k_ex_* = 1 [40]. Nanoparticles obtained using a more flexible microemulsion, such as tergitol/isooctane/water, were successfully reproduced by simulation data using *f* = 30, *k_ex_* = 5 [31].

### 2.3. Initial Concentration inside Droplets

Initially, each kind of reactant (A^+^, B^−^ and R) is distributed throughout the micelles of the corresponding microemulsion according to a Poisson distribution as:

(1) $$P(c_{i})=\frac{{\langle c_{i}\rangle }^{c_{i}}}{c_{i}!}\exp(-\langle c_{i}\rangle ),i=A^{+},B^{-}\mathrm{or}\;R$$

where the number of each reactant per micelle is referred to as *c_i_*, *i* represents one of the metal salts or the reducing agent, and *P*(*c_i_*) is the probability that a micelle carries *c_i_* reactants (A^+^, B^−^ or *R*) whose average occupancy is 〈*c_i_*〉 That is, not all micelles carry the same number of reactants. We present results using different mean values of concentration: 〈c_A+_〉 = 〈c_B+_〉 = 4, 32, 64 and 128 metal ions initially located in a droplet. These values were calculated to simulate concentrations 0.02 M, 0.16 M, 0.40 M and 0.64 M, respectively. Molar concentration was calculated considering the droplet radius *r* of a 75% Isooctane/20% Tergitol/5% water microemulsion (*r* = 4 nm, obtained by DLS). From this radius, and assuming spherical shape (*V_micelle_* = 4/3π *r^3^*), the molar concentration of a micelle containing 64 atoms is calculated from

(2) $$\langle c\rangle =64\frac{\mathrm{atoms}}{\mathrm{micelle}}\frac{1}{V_{droplet}(L.\mathrm{micell}e^{-1})}\frac{1}{N_{\mathrm{AV}}(\mathrm{atoms}.\mathrm{mol}{-}^{1})}=0.4M$$

where *N*_Av_ is the Avogadro’s number. The reducing agent concentration <*c_R_*> was always double that of the average concentration of the metal precursors.

### 2.4. Time Unit Base

The time unit is one Monte Carlo step, defined as follows: One Monte Carlo step starts when 10% of the micelles are chosen to collide at random. Then, micelles fuse and material exchange may take place. The nature and quantity of species inside chosen micelles can be modified according to the exchange criteria described below. Once the composition inside both micelles is updated, one Monte Carlo step (mcs) is completed. The composition inside each micelle is stored step by step, because the sequence of metal reduction is decisive in order to describe nanoparticle structure. We monitored the evolution of particle distribution as a function of time. One simulation run is finished when the composition of every particle inside all micelles remains constant.

### 2.5. Intermicellar Exchange Criterium of Isolated Species (Reactants and Metal Atoms): Intermicellar Exchange Rate

Species can be transferred between micelles during the short-lived dimer formation. This transfer is closely related to the intermicellar exchange rate, because the faster the rate, the more species can traverse the dimer channel during a collision. The exchange criterium is the concentration gradient, that is, species flow from a region (micelle) of higher concentration to one of lower concentration until concentration becomes equal. A simulation parameter *k_ex_* is included to restrict the maximum number of reactants (A^+^, B^+^ or *R*) which can be exchanged between micelles during a collision. In particular, if the higher occupied micelle contains a quantity of molecules greater than *k_ex_*, at the most *k_ex_* reactant molecules can go across the channel towards the micelles containing less reactants. In the case of the number of reactants to be exchanged being lower than *k_ex_*, reactants are redistributed until concentrations inside both colliding micelles becomes equal after collision.

It is assumed that *k_ex_* is mainly determined by the microemulsion composition [24,25,41,42] and material nature is less important. So, although the characteristics of species traversing the channel could modify the intermicellar exchange rate, a single value of *k_ex_* is used in this investigation (*k_ex,_*_A+_ = *k_ex,_*_B+_ = *k_ex,_*_R_= *k_ex_*).

### 2.6. Chemical Reduction Rates

Due to redistribution of material between micelles, both reactants (one metal salt and the reductor) can be located inside the same micelle, so chemical reduction can take place inside the nanoreactor. The reduction potentials of the two metal salts are different, so both chemical reductions will occur at different rates. The two metal salts are reduced according to the simulation parameter v*_i_*, where *i* corresponds to metal salts A^+^ and B^+^. This parameter represents the chemical reduction rate as the probability of obtaining one reduced metal atom from each pair of metal salt–reducing agent molecule available in the micelle. Because of the fastest reduction rate of A^+^, it is associated to v_A+_ = 1, that is, A^+^ reduction rate is instantaneous. This implies that the reaction is completed, and only A atoms and excess of reactants (either A^+^ salt or R) are to be distributed in daughter micelles. Hence reactants A^+^ salt and R will not coexist in a micelle. To consider different reactions rates, the probability of reactants located in the same micelle which reduces to metal atoms can be decreased. Because the final nanoparticle structure strongly depends on the reduction rate ratio, in this study we present results for v_A_ =1 and v_B_ =1, 0.2, 0.1 corresponding to v_A_/v_B_ = 1, 5, 10 respectively. For example, a value v_A_/v_B_ = 5 means that only 20% of the pairs B^+^ salt and a reducing agent available in the micelle produces B atoms. The rest of B^+^ salt and reducing agent, which did not react, remains in the micelle, and can be exchanged or can react in a posterior collision. When both reduction reactions are possible because the three reactants (A^+^, B^+^ or *R*) are located inside the same micelle at the same time, both reductions are allowed to take place during the same collision.

One must keep in mind that the microemulsion dynamics, that is, the whole process of motion-collision-exchange, determines the rate for the exchange of the micelle content. Microemulsion dynamics, which is characteristic of each kind of microemulsion, becomes relevant depending on the relative rates of chemical reaction and intermicellar exchange. The pseudo-phase model is employed when the chemical reaction is very slow compared with the exchange rate. In this case, the reaction “sees” the microemulsion as a static object, so microemulsion dynamics do not have to be taken into account [27]. On the contrary, when the reaction rate is faster than the rate of exchange, this plays a decisive role in the particle formation process [27,43,44]. In this study the reduction rate of one metal is instantaneous, and the second one is a little slower, so both speeds are very fast with respect to the intermicellar exchange rate.

### 2.7. Nucleation

Nucleation is the process by which atoms (ions or molecules), initially isolated in solution, become arranged forming a thermodynamically stable nucleus. The nucleus grows as more atoms are deposited on it. Nucleation is initiated by a random fluctuation which is able to overcome the energy barrier for the phase transition. Once this fluctuation takes place, further growth is energetically favorable. Classical nucleation theory established the existence of a critical nucleus size from which a nucleus can grow instead of dissolving, so a nucleus smaller than the critical size is spontaneously dissolved. In microemulsions, nucleation requires the simultaneous presence inside the same micelle of enough atoms to exceed the critical nucleation size [45]. Nucleation is included in the model by means of the variable critical nucleus n*, which is compared with the actual amount of metal atoms inside the same micelle: if it is smaller than n*, atoms remain free (non-aggregated) inside the micelle because the nucleus is considered to be unstable, and breaks up to produce isolated atoms. This implies that atoms can be exchanged during a posterior collision subject to *k_ex_* parameter. However, if the number of reduced atoms located inside the same micelle exceeds n*, all atoms gather producing a stable nucleus capable of further growth. The exchange of nucleus between colliding micelles depends on the intermicellar channel size, because an aggregate of atoms has to be exchanged as a whole. This kind of material exchange is governed by the film flexibility parameter *f* (see below).

The total Gibbs energy of an atomic arrangement determines the alloying ability of a bimetallic nanoparticle A/B. It depends on composition, because of the different atomic binding energies of A-A, B-B, and A-B species. These different binding energies (A-A, B-B, and A-B) are the cause of the minimum size that must be reached by atoms for nucleation depending on the composition. This phenomena is included in the simulation model by means of the inclusion of three critical nucleus numbers (n_A_*, n_B_* and n_A-B_*). Once the critical number is exceeded, the cluster grows by deposition of all metal atoms (either faster or slower metal) located inside the same micelle. It is assumed that only one particle can be carried by a micelle. That is, only one nucleation event is possible in each micelle.

Previous simulation studies prove that the critical size plays a fundamental role in nanoparticle formation [44,46,47,48], and show that metal segregation not only can be caused by a difference in the reduction rates of the metals but also by a difference in the nucleation rates. In this paper the chemical reduction rates for the two precursors are very similar, so n* was kept constant (n_A_* = n_B_* = n_AB_* = n* =1) to not interfere in the discussion. The combination of larger n* (which implies a delay in nucleation) and almost similar reduction rates would not allow to identify which rate (nucleation or reduction) is the cause of metal segregation in the final nanoparticle. Furthermore, although the fact that n* = 1 applies to both Ag, Pd and Pt is just an approximation, a low n* value can be expected. Due to the very small size of micelles, local concentrations can be very high, favoring nucleation. Ritcey *et al.* [45] propose that reverse micelles constitute environments in which critical nucleus size is smaller than that associated with precipitation in simple aqueous solutions. Within micelles, stable nuclei composed of as few as one, two and four metal ions have been proposed for Co [49], Ni_2_B [50] and CdS [51] respectively.

The surface chemistry is not considered. Pileni [52] demonstrated that the presence of the surfactant layer does not induce a preferential facet growth or truncation in Ag and Cu nanoparticles, that is, surfactant does not play a role during the nanoparticle growth. Different atomic-scale simulations provide detailed information of the surface chemistry of bimetallic nanoparticles of different composition [32,53,54].

### 2.8. Nanoparticle Growth

It is assumed that nanoparticles grow by deposition of metallic atoms on the nucleus, so a growing particle builds up layer by layer. Therefore, whenever a metal atom is located in a nucleated micelle, this atom is deposited on the nucleus. The sequence of metal deposition (A or B) is stored in each micelle at each step.

In addition, nanoparticle can grow by autocatalysis [30,40,48,55]. This kind of growth appears at advanced stages of the synthesis, when collisions between micelles containing reactants and a growing particle simultaneously become frequent. An autocatalytic reduction is simulated by introducing two new criteria: when one of the colliding micelles is carrying a particle, the reduction will occur at double the rate and always on the existing particle. When both colliding micelles are nucleated, reduction takes place in the micelle containing the larger particle, and the produced atom is deposited on it. These criteria allows us to include in the model the fact that a bigger surface (a bigger particle) has a greater probability to play as catalyst. In this way, the growth of a pre-existent nucleus is favored instead of the formation of a new one. To simplify, autocatalytic growth is governed only by particle size, without taking into account the surface composition (A or B).

Another way of growth is Ostwald ripening, *i.e.*, growth of larger particles at the expense of smaller ones by transport of material. Due to the solubility of individual metal atoms in the oil phase not being known, and Ostwald ripening would only be possible if they are soluble, it was proposed that it could be Smoluchowski ripening [48], which concerns diffusion-mediated coagulation of particles. It is not clear which of the two is the dominant process in nanoparticle formation inside micelles, but in any case, it is assumed that the easier solubilization of the smallest particles causes their decrease in size. These atoms/molecules, free in solution, will deposit on the largest particles. That is, large particles grow even larger, drawing material from the smaller ones, which shrink. This possibility is included in the exchange protocol of particles described as follows.

### 2.9. Intermicellar Exchange Protocol of Growing Particles

The transfer of a growing particle to another micelle containing also a particle has to be possible, whenever the channel communicating colliding micelles is large enough. As mentioned before, the size of the channel is represented by the film flexibility parameter *f*, which is strongly correlated to the surfactant film flexibility. Then, a collision between micelles both containing a growing particle will take place in a unidirectional transfer, so that the smaller particle moves always from the initial micelle to the micelle containing the larger one. In this way, two particles in two colliding micelles can give rise to a single particle. This exchange is only possible when the smaller particle can traverse the intermicellar channel, that is, the smaller particle must be smaller than *f* (channel size). This rule is defined simply by the particle size, so the composition (A or B) is not taken into consideration.

All types of material exchange (reactants, isolated atoms and growing particles) are allowed to be exchanged during the same collision.

### 2.10. Droplet Size

Nanoparticle growth may be restricted by the size of the micelles, because the surfactant film covering the micelle has a finite bending modulus. The simulation includes a micelle size parameter, which limits the nanoparticle size establishing a maximum quantity of metal products which can be located inside the same micelle. In this study we present results using low values of reactant concentrations, so that the influence of micelle size on nanoparticle growth is assumed to be negligible.

### 2.11. Describing Metal Distribution in Bimetallic Nanoparticles

Nanoparticle formation finishes when the contents of each micelle do not vary with time. So each simulation run results in a set of micelles, each one of which can contain a particle, whose composition can be different. The metal distribution in the nanoparticle is determined by the order in which the two metals are deposited on the nanoparticle surface. On the assumption that particles are spherical, clustering steps give rise to the build up of concentric layers by adding new atoms to a particle. Therefore, for each nanoparticle, the sequence of metals is stored during the synthesis and divided into ten concentric layers. The number of particles containing each percentage of Au is monitored from the inner layer (core) to the outer layer (shell), so dispersity and averaged composition (% of faster metal A) can be calculated layer by layer. This final distribution is averaged over 1000 runs.

Nanoparticle structure is represented by histograms, in which the layer composition (% of faster reduction metal, A) is represented by a color grading: the degradation goes from blue (0%–10% of fast metal A) to red (90%–100% of A); 50% of each metal is represented by grey. As the color turns lighter, the proportion of pure metal in the layer is higher. The histograms shows how many particles have a given percentage of A in each layer. In this way, histograms allow to analyze the variation of metal arrangements from the early stages (core) as the synthesis reaches the shell formation. The nanoparticle structure is also represented by means of concentric spheres, whose thickness is proportional to the number of layers with a given composition, keeping the same color scheme.

## 3. Results and Discussion

Our hypothesis is that the resulting nanostructure is due to the particular combination of three main factors: the reduction rate ratio between both metals, the amount of metal precursors inside micelles, and the intermicellar exchange rate (determined by microemulsion composition). All together will determine a particular sequence of deposition of the metals, which in turn determines the metal distribution in the final nanoparticle.

### 3.1. Chemical Reduction Rate Ratio

**Figure 1:**
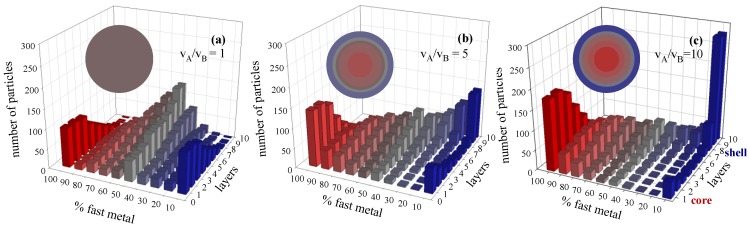
Histograms represent the number of particles with a given percentage of the faster metal A in each layer, from the nanoparticle core to the surface, for different values of reduction rate ratio. (**a**) v_A_/v_B_ = 1; (**b**) v_A_/v_B_ = 5; (**c**) v_A_/v_B_ = 10). Synthesis conditions: film flexibility (*k_ex_* = 5, *f* = 30) and metal salt concentration (〈c〉 = 32 metal salts in a micelle). Scheme color: blue (0%–45% of A), grey (45%–55% of A), red (55%–100% of A). Less red means less A. Circles in each histogram represent the nanoparticle structure in concentric layers, keeping the same color scheme.

It is well-known that different pairs of metals, whose standard reduction potentials are different, show a different metal segregation in a bimetallic nanoparticle. First of all, we will study the modifications in metal distribution in a narrow range of difference between the reduction rates of the two metals. To isolate this dependence, reactant concentration (〈c_A_〉 = 〈c_B_〉 = 32 reactants per micelle = 0.16 M) and intermicellar exchange rate (characterized by *f* = 30, *k_ex_* = 5), must be kept constant. Figure 1 shows the simulation results obtained using different reduction rate ratios. In these figures, the reduction rate of the fast metal (A) is always instantaneous (v_A_ = 1), that is, 100% A reactants inside the same micelle give rise to products. The reduction rate of the slower metal (B) is decreased from v_A_/v_B_ = 1 (100% B reacts, see Figure 1a); v_A_/v_B_ = 5 (20% B reacts, see Figure 1b); to v_A_/v_B_ = 10 (10% B reacts, see Figure 1c). As observed in Figure 1, metal distribution strongly depends on the reduction rate ratio. It is assumed that a large difference in the reduction potential usually results in a core-shell structure and a small difference in the reduction potential leads to an alloy one [18]. Figure 1a shows the metal distribution obtained when the reduction rates are equal (v_A_/v_B_ = 1). It can be observed that the composition of the inner layers (core) is variable: some particles are mainly composed of only one of the metals, and other particles show a core with a different degree of mixture. As the synthesis advances (from the inner to the outer layers), the metal distribution shows a progressive improvement towards a perfect mix of both metals. At the end of the process, the composition of most of the particles is 50% in each metal. That is, nanoparticles show an alloyed structure. When the reduction rate of the metal B is slower (20% B reactants inside the same micelle give rise to products) the structure evolves to an alloyed structure with an A-enriched core and a B-enriched shell, as observed in Figure 1b. Figure 1c shows a core-shell structure, obtained when only 10% B reactants leads to products (v_A_/v_B_ = 10): most particles have a core composed of the faster reduction metal (see red bars on the left), followed by mixed middle layers, and then outer layers composed by the slower metal forming the shell (see blue bars behind on the right). A further increase of reduction rate ratio gives rise to a better metal segregation [29].

**Figure 2:**
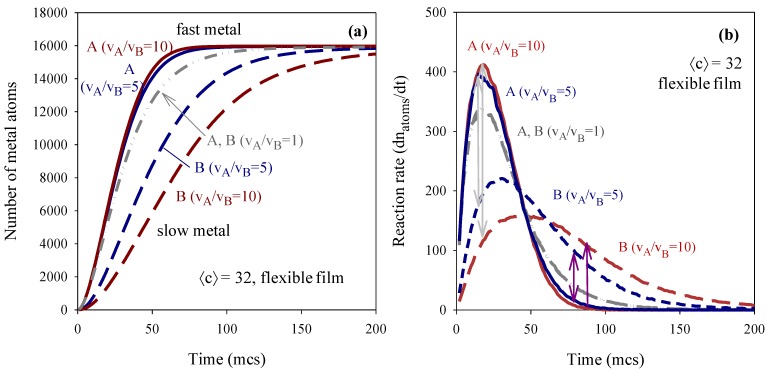
(**a**) Time evolution of the number of metal atoms obtained in micelles using different reduction rate ratio v_A_/v_B_ = 1, 5, 10; (**b**) reaction rate *vs.* time. Continuous and discontinuous lines show the obtaining of fast (A) and slow (B) metals respectively. Synthesis conditions: flexible film (*k_ex_* = 5, *f* = 30), average concentration 〈c〉 = 32 metal salt in a micelle. Scheme color: grey line represents v_A_/v_B_ = 1; blue lines represent v_A_/v_B_ = 5; red lines represent v_A_/v_B_ = 10.

For a better understanding of the chemical kinetics in micelles, the number of metal atoms produced in all micelles was monitored *vs.* time by simulation. Figure 2a shows the number of atoms of A and B (quick and slow reduction products) produced in all micelles as the synthesis advances. This figure corresponds to the same synthesis conditions shown in Figure 1. Continuous lines in Figure 2a represent the number of faster atoms (A) and discontinuous lines correspond to slower ones (B). Blue lines show the case v_A_/v_B_ = 5 and red lines show results for v_A_/v_B_ = 10. Each A curve (continuous line) must be related to the corresponding B curve (discontinuous line in the same color). All curves lead to a plateau when the reactants have been exhausted. The grey dashed-dotted line represents the case v_A_/v_B_ = 1, in which the curves showing the obtaining of faster and slower metals overlap, as expected. When the reduction rates are different (see blue lines, v_A_/v_B_ = 5) the later obtention of the metal which is reduced slower can be clearly observed. As the second metal reduction rate is slowed-down, fast and slow curves appear more separated (see red lines, v_A_/v_B_ = 10). Note that the slopes of all A curves are almost similar. On the contrary, the B curves are strongly dependent on reduction rate.

To gain more insight on how reactant confinement influences chemical reactivity, the reaction rate of each metal was calculated from the slopes of the curves showed in Figure 2a as time proceeds. In this way, both contributions (intermicellar exchange rate and chemical reduction rate) are taken into account to determine reaction rate (dn_metal_/dt). It is important to point out that the chemical reduction rate must be distinguished from the reaction rate. Chemical reduction rates are represented by the simulation parameters v_A_ and v_B_, which are related to the percentage of reactants inside a micelle that gives rise to products during an intermicellar collision. Reaction rates are calculated from simulation results as dn_metal_/dt, so it includes the fact that reaction media is compartmentalized, therefore material intermicellar exchange is also taken into account. Continuous and discontinuous lines in Figure 2b represent the resulting A and B reaction rates, respectively, keeping the same scheme color as used in Figure 2a.

Note that the usual decay *vs.* time is preceded by an increasing rate until a maximum is reached. Then, the usual behavior can be observed: reaction rate decreases as reactants are being consumed in the reaction. The increasing rate before the peak can be explained keeping in mind that the two reactants must be located inside the same micelle before chemical reduction can take place. It implies an intermicellar exchange of material, whose rate is determined by microemulsion composition. In the simulation model, the intermicellar exchange rate is simulated by the parameter *k_ex_*, which quantifies how many ions/molecules can be transferred between colliding droplets. Figure 2 shows data obtained simulating a surfactant film which allows a maximum intermicellar exchange of five reactants (metal salts and/or reducing agent). That is, independently of the metal salt amount inside the droplet, only *k_ex_* = 5 reactants are allowed to be exchanged in each collision. As a result, although an instantaneous reduction is simulated for the faster metal, only a maximum of five A atoms can be obtained during each effective collision. This restriction allows us to understand the A rate profile: at the beginning of the synthesis, three microemulsions are mixed. Each microemulsion is represented as a set of micelles, each one containing one kind of reactant. Micelles collide with each other, but only collisions between one droplet carrying A salt (M-A) and another droplet carrying reductor (M-R) allows the location of both reactants inside the same waterpool. The rest of the possible collisions (M-A and M-A, M-A and M-B, M-B and M-B, M-R and M-R) only redistribute reactants between micelles. Collisions between micelles carrying metal salt B and reductor (M-B and M-R) will be discussed later. When the metal salt A and the reducing agent are located by the same micelle, chemical reduction of A precursor takes place instantaneously. Therefore, as the reactants are redistributed, more collisions will be effective, providing metallic A atoms and increasing the reaction rate. One can conclude that the speed at which the maximum rate is achieved only depends on the material intermicellar exchange rate. That is, compartmentalization of reaction media causes the faster reduction to be mainly controlled by the intermicellar exchange rate. As a consequence, all slopes at the beginning of A curves in Figure 2b (see continuous lines) are equal. As redistribution of reactants enables the reactants to encounter and A chemical reduction takes place, reactants are been consumed, resulting in a decreasing reaction rate. As shown in Figure 2a, the reaction rate profile of the faster metal is almost the same for the three values of v_A_/v_B_.

In relation to the reaction rate of the slower metal, discontinuous lines in Figure 2b show that it is strongly dependent on reduction rate ratio, even though the intermicellar exchange rate is the same for both reactants (*k_ex_* = 5). B reduction rate is 5/10 times slower than A reduction rate. That is, only 10% (v_A_/v_B_ = 10) or 20% (v_A_/v_B_ = 5) of reactants (B salt and reducing agent) located in the same droplet will react to produce B metal atoms in each collision. Therefore, because of the intermicellar restrictions, only *k_ex_* = 5 reactants can be transferred between droplets, and in addition, only a percentage of reactants inside the same micelle gives rise to products. Consequently, the slopes of the discontinuous lines (slower metal B) in Figure 2b before the maximum, decrease as the chemical reduction rate of B metal is slower. Furthermore, as the B chemical reduction diminishes, the reaction rate maximum is lower and appears at later stages of the synthesis, as expected (compare blue and red discontinuous lines). It is interesting to note that, after the maximum, the slope of B reaction rates strongly depends on v_A_/v_B_ (in contrast with A slopes). This can be understood by keeping in mind that at the advanced stages of the synthesis, reactants have been exhausted, so that B reaction rate continuously decays, and the rate of this decay will be faster as the reactants are consumed faster.

Reaction rates profiles can be directly related to nanoparticle structure. At the beginning of the synthesis, when the core is forming, the gap between the faster and the smaller reaction rates is higher at a higher v_A_/v_B_ ratio, giving rise to a higher A-enrichment in the core (see grey arrows in Figure 2b). Likewise, at advanced stages of the synthesis, when A salt is almost consumed, the gap is larger if v_A_/v_B_ ratio is larger (see pink arrows in Figure 2b). This results in a B composed shell, which is better separated as the v_A_/v_B_ ratio increases.

### 3.2. Metal Salt Concentration

**Figure 3:**
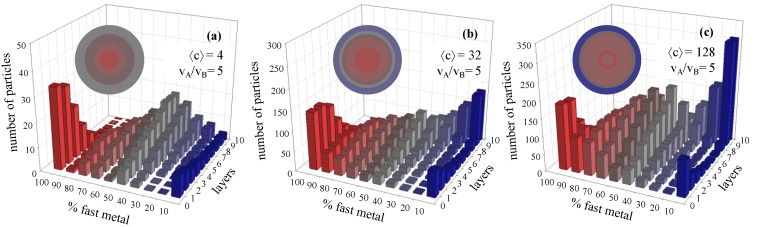
Histograms represent the number of particles with a given percentage of the faster metal A in each layer, from the nanoparticle core to the surface. Synthesis conditions: reduction rate ratio v_A_/v_B_ = 5; film flexibility (*k_ex_* = 5, *f* = 30). (**a**) Metal salt concentration 〈c〉 = 4 metal ions in a micelle; (**b**) 〈c〉 = 32 metal ions in a micelle; (**c**) 〈c〉 = 128 metal ions in a micelle. Scheme color: blue (0%–45% of A), grey (45%–55% of A), red (55%–100% of A). Less red means less A (faster metal). Circles in each histogram represent nanoparticle structure in concentric layers, keeping the same color scheme.

To study the dependence of nanoparticle structures on metal salt concentration, a small difference between both chemical reduction rates (v_A_/v_B_ = 5) was chosen in order to find out if the nanoparticle structure can be modified by a change in reactant concentration under these conditions. Figure 3 shows the histograms obtained keeping the reduction rates ratio (v_A_/v_B_ = 5) and film flexibility (*f* = 30, *k_ex_* = 5) constant, while the metal salts concentration is increased (see Figure 3a–c). It can be observed that, in spite of the small difference in chemical reduction rate, the metal distribution depends on the metal salt concentration. One can observe that a very small concentration (see Figure 3a, 〈c〉 = 4 metal ions in a micelle) lead to an A-enriched core covered by an alloyed shell. A higher concentration gives rise to the incipient formation of a shell (see Figure 3b), which is more enriched in the slower product with the increase of concentration (see blue bars on the right in Figure 3c).

**Figure 4:**
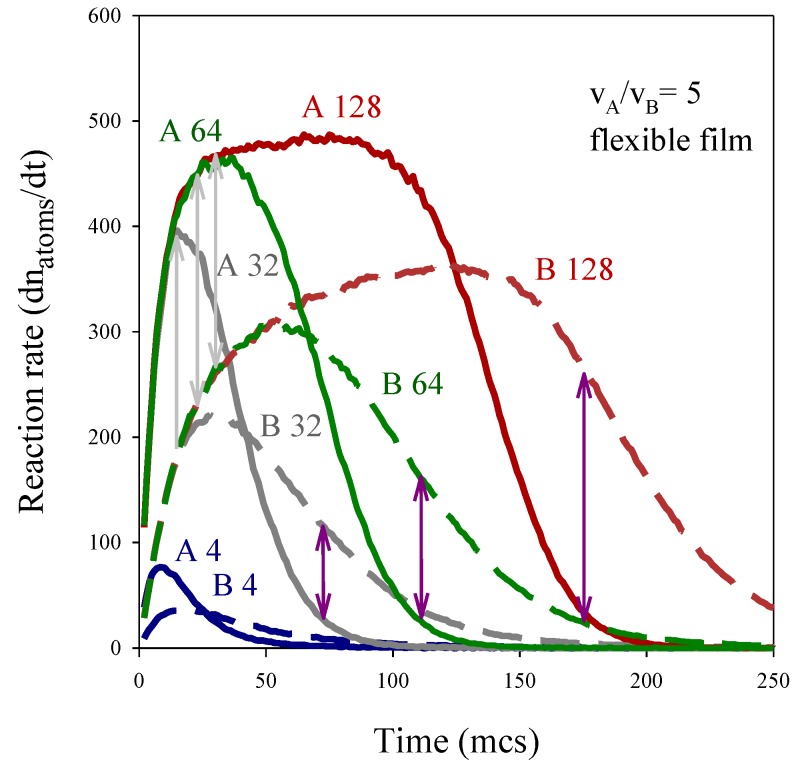
Reaction rate *vs.* time. Continuous and discontinuous lines show the reaction rate of fast (A) and slow (B) metals respectively. Synthesis conditions: reduction rate ratiov_A_/v_B_ = 5; flexible film (*k_ex_* = 5, *f* = 30). Metal salt concentration: 〈c〉 = 128, 64, 32 and 4 metal salt in a micelle are represented by red, green, grey and blue lines respectively.

These nanostructures can be easily understood by analyzing the relative reaction rates. Figure 4 shows the reaction rates calculated as dn_metal_/dt, for the same synthesis conditions as those used in Figure 3. In all cases it can be observed that the greater the concentration, the faster the reaction rate, as expected. However, the behavior of the faster and the slower metal with increasing concentration is different. Firstly, we will focus our attention on the dependence of A reaction rate on concentration (see continuous lines in Figure 4). Figure 4 shows that the slope of the fast metal rate is the same for all concentrations and seems to reach a threshold from which it cannot increase anymore. As mentioned, A reaction rate is controlled by exchange rate, so the faster reaction rate increases as quickly as the material intermicellar exchange allows the localization of both reactants inside the same droplet. Therefore, while reactants are being redistributed between micelles, the reaction rate increases, until it starts to diminish as the reactants are exhausted. So larger values of the reaction rate are reached by increasing A salt concentration, as expected. If the concentration is high enough, the faster reaction rate achieves the intermicellar exchange rate (see the plateau in Figure 4, red line) and it remains constant while there is A precursor, even though the A amount inside the micelles would be larger. If the A salt concentration is small, the reaction rate does not reach intermicellar control because A salt is exhausted earlier. Summarizing, the speed at which the intermicellar control is achieved does not depend on A salt concentration, but it only depends on the material intermicellar exchange rate (see equal slopes at the beginning of Au curves in Figure 4). Likewise, according to model predictions, the decrease in rate is not influenced by the A concentration either, because, once the A salt is redistributed, Au reduction only depends on the intermicellar rate: it steadily diminishes while there is A precursor. Finally, it is interesting to emphasize that if synthesis conditions lead to the plateau achievement, the classical belief that the larger the reactant amount, the faster the reaction rate is not valid in this confined media.

In relation to the slower reaction rates, different behavior before and after the peak can be observed. Before the peak, the reaction rates at different concentrations increase with the same slope, that is, the speed at which the maximum is reached does not depend on concentration. The reason is that, independently of concentration inside the micelle, only 20% of the *k_ex_* = 5 exchanged reactants can react during each collision. Because of the intermicellar exchange control, the only effect of increasing concentration is the achievement of higher and later maxima, as expected. However, the behavior after the peaks in Figure 4 shows an unexpected result: the slopes of B reaction rates strongly depends on concentration, in contrast with A reaction rates. One could expect that B reaction rate after the peak would not depend on concentration because the restrictions are the same before and after the peak. So *a priori*, B reaction rate would uniformly decrease while there is B precursor. The main difference in chemical kinetics between the faster and the slower metal is the effect of confinement on each metal. In the case of the faster metal, once both reactants are located inside the same droplet the reaction rate is very quick. On the contrary, if the reduction rate inside the micelle is slow, many reactants do not react, stay in the micelle, and can be exchanged or can react later. This leads to a local accumulation due to a “cage-like” effect. Briefly, the cage effect assumes that encounters between reactants take place in a different way in a solution than in a gas. In a solution, a reactant molecule enclosed in a solvent cage undergoes many collisions with the solvent molecules surrounding it before it escapes from the cage. The reactant molecules will move from cage to cage in the solvent matrix, so the two reactants will eventually find themselves in the same solvent cage. They can fail to react the first time, but several collisions may occur as long as reactants remain in the same solvent cage. That is, there will be fewer encounters but they will be close together for much longer than in a gas. However, if a chemical reaction takes place in gas phase the molecules have the freedom to go anywhere, so many collisions between reactants take place. However, if the collision should fail to react, the reactants move away and are unlikely to meet again anytime soon. So there will be more encounters between reactants, but they will be together a shorter time. Micelles were previously described as “supercages” [56], and many studies proved a cage-effect in chemical reactions in micelles [25,57,58]. The hypothesis is that this approach can be used to compare the chemical kinetics in micelles and in a solution, instead of in liquid and in gas phases.

The fact that a micelle plays the role of a cage, does not affect the fastest reduction, because it is considered instantaneous. That is, when the exchange of material between colliding droplets results in the reactants A^+^ salt and R being located in the same micelle, all of them produce A metal atoms without delay. Therefore, after interdroplet collision, only A atoms and the excess of reactants (either A^+^ salt or reducing agent R) are contained in the micelle. In other words, reactants A^+^ salt and R cannot coexist inside the same micelle. On the contrary, slow reactant confinement will strongly affect the slow reduction rate. A value v_A_/v_B_ = 5 means that only 20% of the pairs B^+^ salt and R available in the micelle produces B atoms. The rest of B^+^ salt and reducing agent, which did not react, remains in the micelle and can be exchanged or can react later. That is, the pairs B^+^ salt and R can fail to react the first time, but they have more opportunities as long as they remains in the same micelle. Therefore, slow reduction proceeds while there are enough reactants inside a micelle, without having to depend on a new intermicellar exchange. This means that the amount of pairs of reactants B^+^ salt and R inside the micelle, available to react, is much higher than the exchanged pairs during the last collision. As a consequence, B reaction rate not only depends on the exchange rate (as the case of A metal) but also on the reactants accumulation. It results in a faster B reaction rate due to the cage effect. This cage-like effect does not concern fast metal reduction, because A reduction is instantaneous so A precursors are not accumulated.

Cage-like effect takes place to a greater extent at high concentrations, because more reactants lead to more accumulation of B. The different decay of B reaction rate can be accounted for by the cage effect: After the maximum, when the B reaction rate continuously decays, the rate of this decay will be faster as the reactants are consumed faster. If the cage-like effect is more pronounced at higher concentration, it also results in a quicker decay of B reaction rate.

Finally, the resulting nanostructures can be explained on the basis of reaction rate profiles. The main feature is the formation of a B-shell when the concentration is large (see Figure 3). The outer layers will be composed by B if A reduction is almost finished but B reduction rate is still high. Pink arrows in Figure 4 show the difference between both reaction rates at each value of concentration towards the end of the process (when A rate is close to zero). It is noteworthy that the difference between both rates increases with concentration. This results in the formation of outer layers whose B enrichment is higher as concentration is larger. On the contrary, the differences between A and B rates during the early stages, when the core is forming, are negligible. Grey arrows in Figure 4 show the gap between the maximum A rate and the B rate at different concentrations. One can observe that the length of the arrows does not depend on concentration, which can be related to the almost similar core enrichment shown in Figure 3.

## 4. Conclusions

Monte Carlo simulations have been employed to determine the mean structure and the reaction rates of bimetallic nanoparticles prepared via a microemulsion route. On the basis of the nanoparticle, build up by bringing together new layers, the sequence of metal reduction defines the resulting metal distribution. The combination of three factors (intermicellar exchange rate, reduction rate ratio between both metals, and metal salts concentration inside micelles) determine the reaction rates of the metals, which in turn define a particular sequence of deposition of the metals. The rates of reaction of each metal were calculated for a particular microemulsion composition, characterized by a fixed intermicellar exchange rate. Simulation results allow us to conclude that reactant confinement is a factor of critical importance in the metal reduction reaction in micelles. Model predictions show that, even in the case of a small difference in reduction potentials of both metals (about 0.15 V), the formation of a shell in a bimetallic nanoparticle can be manipulated solely by varying concentration: a pure core with a mixed surrounding shell is obtained using low concentrations, and a core-shell structure is obtained at higher concentration. This modification of metal arrangement with concentration is analyzed from a mechanistic point of view, proving that it is due to the different impact of confinement on each metal: the reaction rate of the faster metal is only controlled by the intermicellar exchange rate, so it cannot be accelerated by an increase in concentration. On the contrary, the slower metal reduction is also affected by a cage-like effect, which is more pronounced as the concentration is higher.

In this paper we have shown how the Monte Carlo simulations approach can help to identify suitable synthesis parameters which influence the metal segregation in a bimetallic nanoparticle. These results may open a new door by which the experimental conditions for designing specific bimetallic structures can be tuned.
